# VMCMC: a graphical and statistical analysis tool for Markov chain Monte Carlo traces

**DOI:** 10.1186/s12859-017-1505-3

**Published:** 2017-02-10

**Authors:** Raja H. Ali, Mikael Bark, Jorge Miró, Sayyed A. Muhammad, Joel Sjöstrand, Syed M. Zubair, Raja M. Abbas, Lars Arvestad

**Affiliations:** 1 KTH Royal Institute of Technology, Swedish e-Science Research Centre, Science for Life Laboratory, School of Computer Science and Communication, Solna, SE-171 77 Sweden; 2 KTH Royal Institute of Technology, School of Information and Communication Technology, Kista, SE-164 40 Sweden; 3 Department of Numerical Analysis and Computer Science, Swedish e-Science Research Centre, Science for Life Laboratory, Stockholm University, Stockholm, SE-100 44 Sweden; 4 KTH Royal Institute of Technology, Laboratory for Communication Networks, School of Electrical Engineering, Stockholm, SE-100 44 Sweden; 5 Department of Computer Science and Information Technology, University of Balochistan, Quetta, PK-87 300 Pakistan; 6 Department of Computer Science and Engineering, University of Gothenburg, Gothenburg, SE-411 37 Sweden

**Keywords:** Convergence, Markov chain Monte Carlo, Metropolis-Hastings, Phylogenetics, Software, Visualization

## Abstract

**Background:**

MCMC-based methods are important for Bayesian inference of phylogeny and related parameters. Although being computationally expensive, MCMC yields estimates of posterior distributions that are useful for estimating parameter values and are easy to use in subsequent analysis. There are, however, sometimes practical difficulties with MCMC, relating to convergence assessment and determining burn-in, especially in large-scale analyses. Currently, multiple software are required to perform, e.g., convergence, mixing and interactive exploration of both continuous and tree parameters.

**Results:**

We have written a software called VMCMC to simplify post-processing of MCMC traces with, for example, automatic burn-in estimation. VMCMC can also be used both as a GUI-based application, supporting interactive exploration, and as a command-line tool suitable for automated pipelines.

**Conclusions:**

VMCMC is a free software available under the New BSD License. Executable jar files, tutorial manual and source code can be downloaded from https://bitbucket.org/rhali/visualmcmc/.

**Electronic supplementary material:**

The online version of this article (doi:10.1186/s12859-017-1505-3) contains supplementary material, which is available to authorized users.

## Background

Bayesian inference using Markov chain Monte Carlo (MCMC) is today a common and trusted approach in molecular phylogenetics (see, e.g., in [1–4]), and is used by phylogeny inference software such as MrBayes [5], BEAST [6], BAli-Phy [7], PrIMe [8] and JPrIME [9]. A typical goal in phylogenetics is to determine evolutionary relationships, for a set of species or for genes of interest, but researchers may also be interested in other parameters, for example parameters related to substitution patterns or duplication/loss processes. An advantage with Bayesian phylogenetic inference is that you can obtain posterior distributions of evolutionary parameters, conditional on your data, where the evolutionary parameters can be classified as discrete parameters (e.g., phylogenetic trees) or continuous (e.g., duplication and loss rates) [10]. For phylogenetics, Bayesian inference is often complicated by the continuous parameters’ dependency on discrete parameters such as trees, that due to their structure can have problems with mixing.

The output from MCMC, the trace, is a conditional sampling of unknown parameters, needing post-processing to yield the desired posterior distributions. Users need to inspect the trace for possible non-convergence, estimate burn-in (how many of the first samples should be discarded?), assess mixing (does it look like a random sampling of the posterior?), and compute parameter statistics. Post-processing can, however, be constrained by practical difficulties, in particular due to presence of both discrete and continuous parameters in the trace, and due to the need to manually extract useful information from MCMC runs, currently using multiple software (for example Tracer [6], AWTY [11] and CODA [12]). User-friendly interfaces supporting graphical and interactive parameter exploration currently has potential to improve.

When converged, MCMC samples are drawn from the underlying stationary distribution and the trace represents a sample from the posterior distribution of analyzed parameters. After removing burn-in samples, a converged chain displays little correlation between remaining samples, which also indicates good mixing. Convergence is, in theory, guaranteed in MCMC if the chain is allowed enough iterations. However in practice, it is not possible to estimate how many iterations are needed, nor is it possible to determine whether the chain has converged. Hence, heuristics are used to assess non-convergence and the initial “burn-in”. Software like CODA [12], AWTY [11], and Tracer [6] have support for this, and more, and for example MrBayes [5] has a convergence diagnostic based on standard deviation of split frequencies built in. These heuristics analyze a single parameter of the chain at a time and the decision of convergence is, usually, left to visual analysis of the trace and the heuristic value proposed for the parameter. For example, Tracer [6] suggests the heuristic that the effective sample size (ESS) [13] is greater than 200, for all parameters, as well as manual analysis of the trace to ascertain that the chain has indeed converged. Please note that the recommendation/heuristic of ESS greater than 200 by Tracer does not have a theoretical justification or a systematic study to support it and using a sufficiently large ESS for assessing convergence for high-dimensional MCMC is recommended by Gong and Flegal [14]. In the convergence analysis of large data sets, e.g., for genome-wide analysis, the manual inspection of convergence becomes a bottleneck and one would like to rely on automated non-convergence assessment and burn-in estimation based on multiple convergence diagnostics. Therefore, we see a need for simplified large-scale convergence analysis.

There are several reasons to scrutinize individual traces. First, one may doubt automatic convergence/burn-in assessment, e.g., when different convergence diagnostics give contradictory assessments. Second, there may be doubts regarding the MCMC proposals, in particular, when the sampled posterior distribution is multimodal and seemingly converged multiple runs provide very different samples. Third, there may be questions regarding mixing of an MCMC chain, e.g., when a trace is stuck in a local optimum for a long time and the proposal acceptance ratios of parameters are low. Fourth, surprising results, e.g., when a new result contradicts previous studies, a trace should be scrutinized to establish that the MCMC has not behaved strangely. Mixing of parameters is an important aspect of a successful MCMC run, in particular for phylogeny analysis where one wants to make sure that the tree posterior is sampled well. Although trace statistics can give important indications, various visualizations are often needed to better understand the different aspects of a chain and its trace. We have experimented with visualizations that focus on trees and their relation to the trace, that we have not found in Tracer, AWTY, and CODA, and believe the community can benefit from them.

We present VMCMC, a tool for phylogenetic MCMC analysis, with support for analysis and exploration of chain convergence, burn-in estimation, trace visualization, parameter estimation, graphical display of parameter traces, which can run both as a command-line tool and as an application with a graphical user-interface (GUI).

## Implementation

VMCMC presents MCMC traces statistically and visually, and enables both automatic and interactive analysis. VMCMC also supports output from many popular MCMC programs, e.g., PrIMe, JPrIME, BEAST, and MrBayes.

There are three use cases for VMCMC that may be of particular general interest:

- **Large-scale automated analysis:** Applying MCMC on genome-wide data, with perhaps hundreds or thousands of phylogenies to estimate, users typically make arbitrary burn-in decisions and, e.g., throw away the initial 25% of a trace. VMCMC can be used on the command line, easily integrated in scripts, and can be asked to estimate a burn-in, assess non-convergence, and estimate parameters automatically.
- **Detailed trace exploration:** When trying to understand a single MCMC trace, and decide whether it is plausible that it has converged, it can be illuminating to see where in a trace certain trees are found, determine majority rule consensus trees and other parameters for different parts of a trace, and have tree space visualized. VMCMC supports a high degree of interactivity for such exploration.
- **Simultaneous handling of both real and tree parameters:** Currently, there is no software that simultaneously analyzes both tree and continuous parameters of a chain. The available MCMC analysis software either handle the continuous parameter (CODA and Tracer) or the tree parameter (AWTY) but not both simultaneously. VMCMC supports analysis of both types of parameters simultaneously and can, e.g., show the occurrence of a specific tree topology in the trace of a real parameter or show the effect of changing burn-in on both the real and tree parameter simultaneously.

### Features

**Fig. 1:**
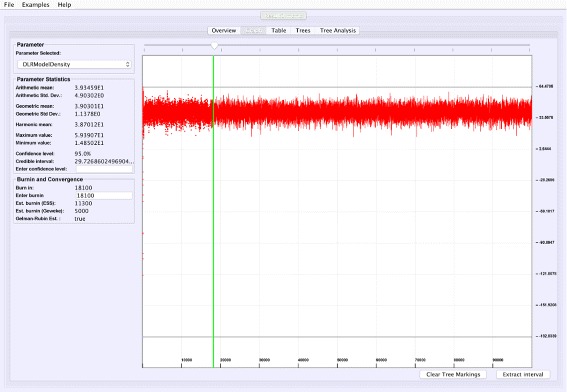
Graphical trace visualization. The figure depicts the “Graph” tab of VMCMC from analysis of a typical MCMC trace obtained from JPrIME by using tetrapod dataset “DS1” collected by Hedges et al. [25]. The trace of the “DLRModelDensity” parameter is shown, which appears to be converged (the difference between values of initial few samples and later samples) and also shows good mixing (fuzzy caterpillar-like trace). The *left* panel shows the selected burnin, the parameter statistics after removing the burn-in, and convergence diagnostics from three standard methods. The panel also provides the option to zoom in to the trace by selecting a portion of the trace and then pressing the “Extract interval” button to see the behaviour of a chain in the selected interval
- **Tree analysis**: VMCMC presents a posterior distribution of trees, sorted by probability and hence easy access to the tree with maximum *a posteriori* probability and the closest alternatives. Selected trees are shown in Newick format and are visualized (Fig. 2) in a panel using code from forester [15]. A majority rule consensus tree can be computed, also restricted to selected trees, thus simplifying comparative tree analysis.

Like AWTY, Tracer, and CODA, VMCMC can also be used for graphical trace visualization, standard parameter statistics, and posterior distribution estimation. Some features unique to VMCMC are given below:

- **Graphical trace visualization**: VMCMC employs a user-friendly GUI to display the trace of a selected real parameter, which helps in seeing trends for a particular parameter as well as visually inspect whether this parameter may have converged (Fig. 1).

**Fig. 2:**
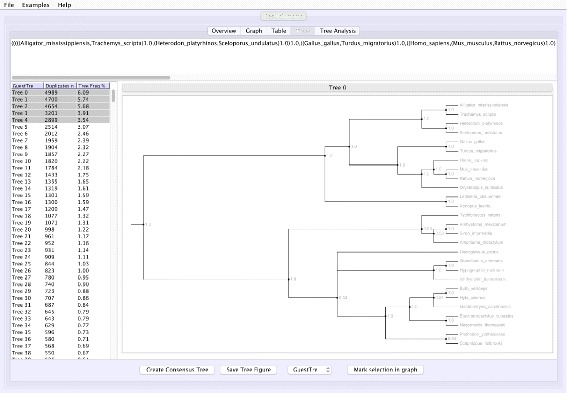
Screen shot of the “Tree” tab in VMCMC for an MCMC trace obtained from JPrIME on the tetrapod dataset “DS1” collected by Hedges et al. [25] The *left* panel shows the estimated posterior on trees sorted in descending order of probability, after removing the burn-in selected in the “Graph” panel. The *right* panel displays either the selected tree or (as in this figure) a consensus tree of multiple selected trees with support values for each edge. The *top* panel shows the displayed tree in Newick format. The presented consensus tree has five nodes where the top five trees differ. Note that support values are determined only by the selected trees and their probabilities
- **Tree space analysis**: VMCMC calculates unweighted Robinson-Foulds distance [16] between each pair of trees and displays a two-dimensional projection (Fig. 3) using a multi-dimensional scaling technique [17–19], where similar trees are expected to cluster. Point sizes proportional to probabilities inform the user of the estimated posterior.

**Fig. 3:**
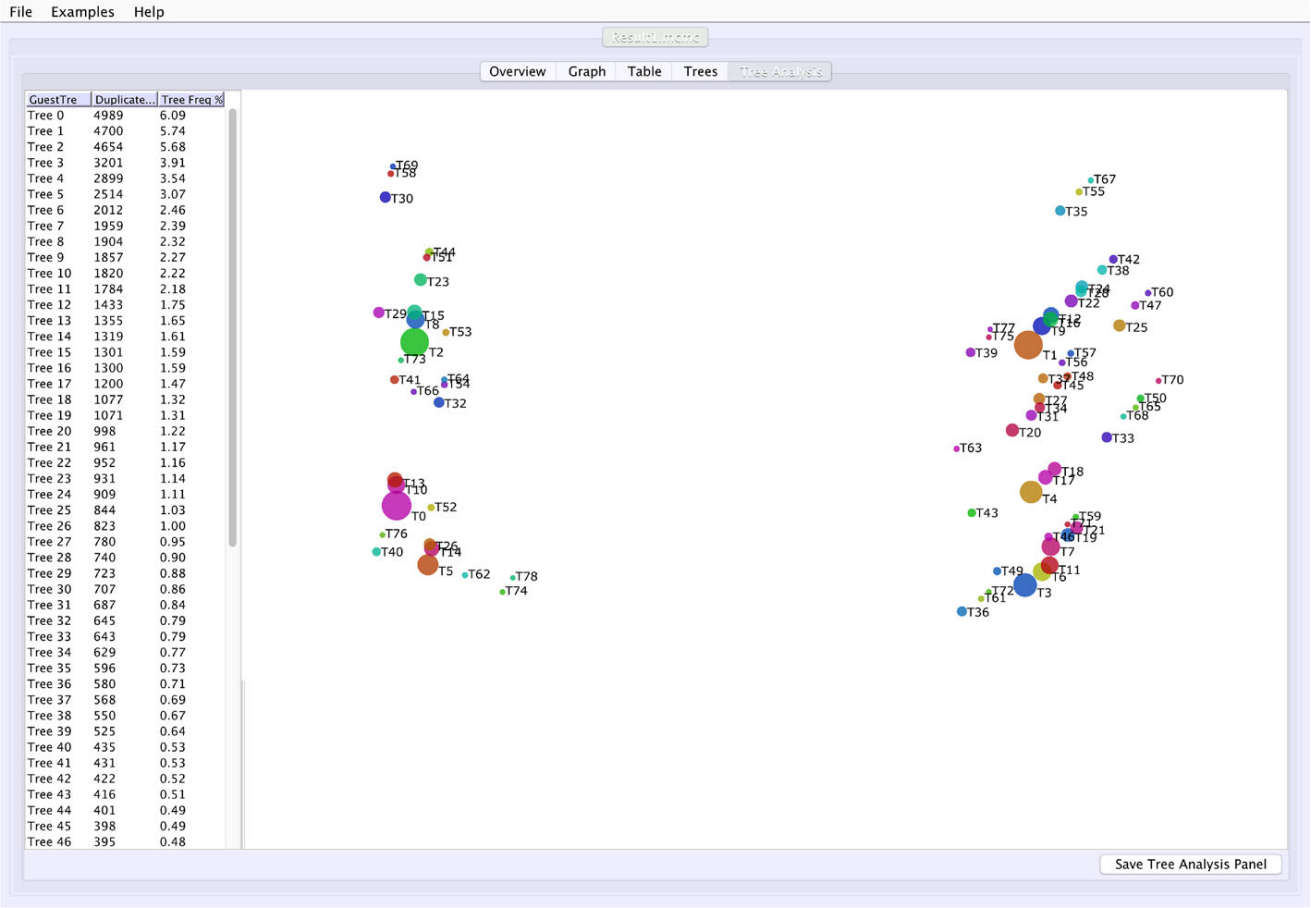
Screen shots of Tree Analysis tab of VMCMC for an MCMC trace obtained from JPrIME on the tetrapod dataset “DS1” collected by Hedges et al. [25] The figure shows the “Tree Analysis” tab for the trace given in Additional file 3. The *right* panel visualizes pairwise distances between trees in the posterior distribution using a multi-dimensional scaling technique [19]. The panel can be used to assess mixing for the tree parameter and to visualize groups of trees that have been traversed during the run. *Circle* sizes reflect posterior probability and color has no significance other than to improve legibility. In this example, T1 seems to be distant from T0 and T2, and the chain appears to be sampling from these trees and trees around them without remaining on intermediate trees. This indicates, in agreement with Whidden et al. [27], that the DS1 dataset has a bimodal distribution. Presence of many trees in the vicinity of T0, T1, and T2, also indicates that the chain is mixing well for the tree parameter
- **Visualizing tree mixing and parameter dependence**: VMCMC can indicate where a selected tree is found in the trace, thus help visualizing tree mixing and how parameters and trees are correlated (Fig. 4).

**Fig. 4:**
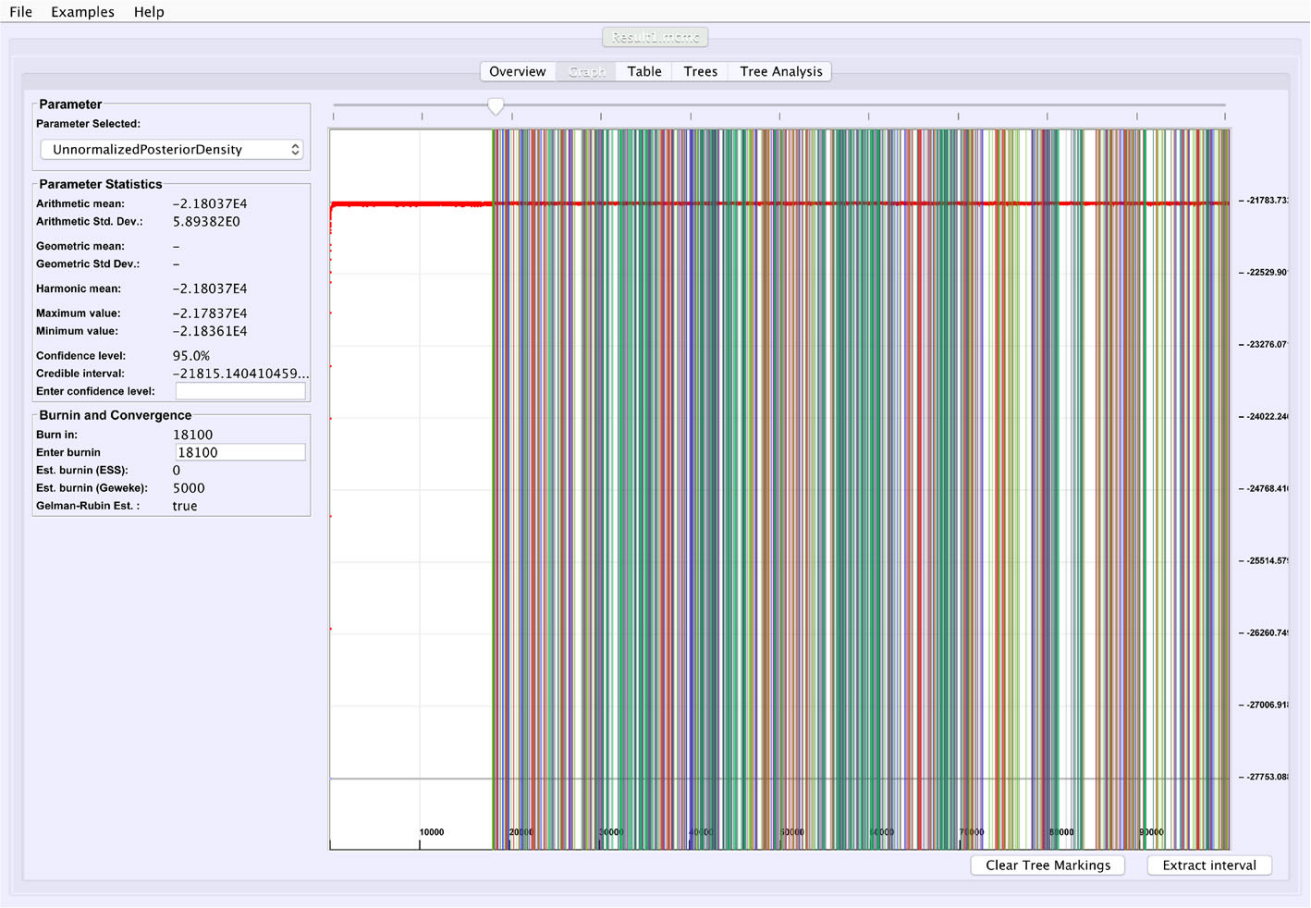
Visualizing tree mixing and parameter dependence: We go back to the Graph tab after selecting the top five trees in Fig. 2 and clicking “Mark selection”. The trace plot is then colored where the selected trees appears in the trace, which is a means of to visualize mixing and to determine the dependency between continuous parameters and trees (the trace plot may be hard to see in the image due to dark colors in print, but the colors are lighter and semi-transparent on screen). For the example trace (in Additional file 3), frequent change of color and no long stretches of a single color is indicative of good mixing for the tree parameter
- **Convergence and burn-in**: Convergence assessment and burn-in estimation can be performed using Geweke’s convergence diagnostic [20], Gelman-Rubin’s convergence diagnostic [21], and Höhna-Sahlin’s ESS-based estimator [22, 23].
- **Analyzing parallel chains**: Currently, traces from two parallel chains can be analyzed and visualized together (Fig. 5). Convergence can be tested on numerical posteriors by applying a Mann-Whitney U test, and tree split distributions [24] can be compared using a chi-square test for two samples. VMCMC provides the flexibility to perform parallel chain analysis using user-specified burn-ins for both chains. The parallel chains are appended to each other after removing specified burnins for both chains, and statistics and posterior distribution is calculated for joint traces. This is a useful feature in cases with poor convergence or high computational demands.

**Fig. 5:**
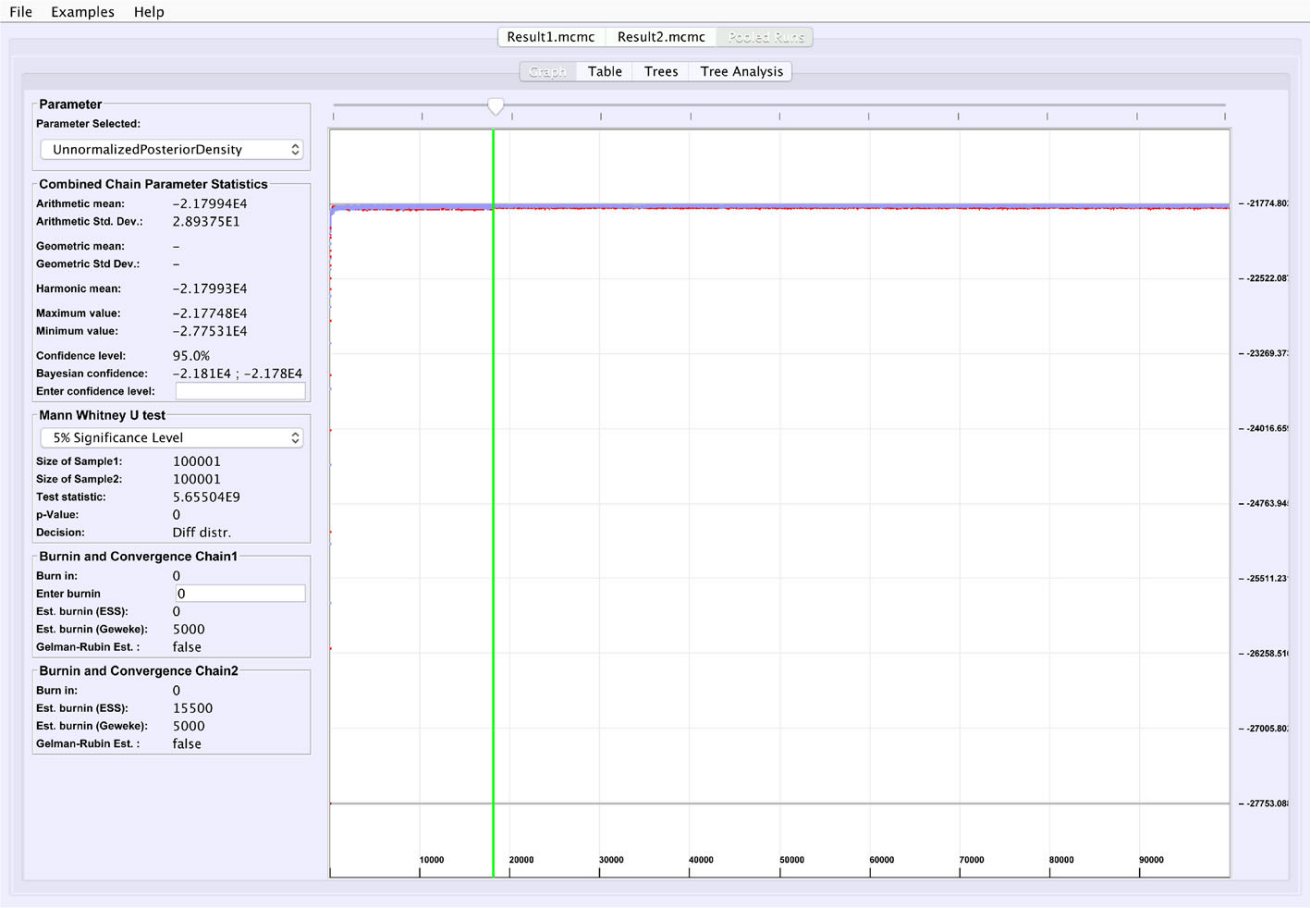
Parallel Chain Analysis. The figure shows the super-imposed traces of a selected parameter for two parallel chains on the same dataset. VMCMC uses a Mann-Whitney *U* test for the real parameters (details seen in the *left* panel) and a chi-square test for two samples for the tree parameter to assess if both the chains have converged to the same distribution. For the example dataset DS1, both chains (whose traces are given in Additional files 2 and 3) appear to be sampling from different distributions
- **Command line tool**: VMCMC can be used both as a GUI application and as a command line tool suitable as a component when writing scripts. Command line output is given in JSON format for easy parsing.

VMCMC tries to hide the computational costs inherent in the problem and utilizes multithreading to perform computations (e.g., in loading of graphics, and in calculation of statistics and of convergence diagnostics) independently from the user interface. Some calculations are delayed: the “Tree Analysis” tab is not prepared until the user asks for it to avoid unnecessary calculation of the needed, and expensive, two-dimensional Robinson-Foulds distance matrix. Furthermore, in order to show trees with non-negligible posterior frequency only and to speed up the loading of analysis on “Tree Analysis” tab, we have limited the computation of distance matrix to unique trees with posterior probability at least 0.2%. While VMCMC can work with any number of unique trees in the posterior, a warning for expecting a delay in loading the tab is generated if the posterior contains more than 45 unique trees with frequency greater than 0.2%.

We have applied VMCMC to real datasets and experienced response times that we feel are reasonable given the trace sizes. Table 1 displays the system time taken by VMCMC from specifying the input file to loading of graphical results on a standard MacBook Pro with 2.6 GHz Intel Core i5 processor and 8 GB RAM.

**Table 1:** Execution time from specifying the input file to presentation of graphical results in VMCMC

Size of tree	Posterior	Trace size	Time
(#leaves)	(# trees)	(# samples)	(seconds)
9	20	10^5^	3.3
12	27	10^3^	2.2
42	105	10^5^	4.2
185	267	10^5^	14.0

The amount of time taken by VMCMC from input to display of results is proportional to the number of samples and the size of the trees. For average sized trees, the loading delay of “Tree Analysis” tab is almost negligible but for large trees, the delay becomes noticeable

## Results and discussion

For an example application of VMCMC, we used a tetrapod 18S ribosomal RNA dataset, named “DS1”, collected by Hedges et al. [25]. DS1 is known to be problematic for convergence and mixing of the tree parameter [26, 27]. We ran JPrIME twice with the same settings (using the JC69 model and default parameters; the species tree was dated using TimeTree.org [28] and is available as Additional file 1), each time for 10 million iterations and sampling every 100 iterations, yielding two traces containing 10^5^ samples which we name *trace 1* and *trace 2* (available in Additional files 2 and 3). Here we present how VMCMC can be used to evaluate mixing of the tree parameter for trace file (Additional file 3). The tutorial for VMCMC is available as Additional file 4.

Figure 1 shows the trace plot of *DLRModelDensity* parameter (this is the probability reported from the DLR submodel [8]) for trace 1, where the fuzzy caterpillar-like trace indicates that the trace does not show non-stationary behavior for *DLRModelDensity* parameter (i.e., the chain does not seem to be “stuck” in a state, nor still continuously improving). In the “Tree” tab, we see a summary of the tree posterior on the left. As an example, we have selected the top five (by frequency) trees and computed a majority-rule consensus-tree for them (Fig. 2). The consensus tree has five edges with support values lower than 1.0, indicating where the selected trees differ. Looking at the “Tree analysis” tab (Fig. 3), the size of each topology (represented by a colored circle) is proportional to the frequency of each topology in the trace. The “Tree analysis” tab also shows that the MCMC chain is repeatedly sampling T0 and similar trees (e.g., T2 and T5), or T1 and similar trees (e.g., T3 and T4), but the transition to other regions is rare. The clear partitioning of tree space into two disjoint parts is indicative of a bimodal distribution. This conclusion is in agreement with previous studies [26, 27] that suggest the data is bimodal. If we select the five top trees, push the “Mark selection in graph” button, and view the “Graph” tab again (Fig. 4), the regions where the selected trees are found in the trace are marked in different colors. We observe intermixing of all five colored lines and no long undisturbed stretches of a single color.

Comparing two parallel chains, run independently on the same data, is another way to investigate MCMC mixing. VMCMC can load two traces and superimpose plots. Figure 5 displays superimposed trace plots, from the DS1 dataset, where one can see that the red trace is different from blue trace at many places. Such aberrations are also checked through statistical hypothesis testing, and we note that the Mann-Whitney U test (left sidebar in Fig. 5) rejects the hypothesis that the traces are from the same distribution and a two-sample chi-square test on trees’ split frequencies indicates the same (not shown here). We conclude that the two parallel chains are sampling from two different posterior distributions and have either not reached the stationary distribution, or need more iterations to be able to safely conclude convergence.

VMCMC has implemented various convergence diagnostics commonly used by phylogenetics community, e.g., Gelman-Rubin’s convergence diagnostic [21], effective sample size (ESS)-based diagnostics and Geweke convergence diagnostic [20]. Please note that some of these diagnostics are outdated and these will either be removed or be replaced in the newer releases of VMCMC by more accurate and recent diagnostics like Fixed-Width Stopping Rule (FWSR) [14]. To elucidate this point, Gong and Flegal [14] have shown that Geweke convergence diagnostic [20] is misleading and outdated for assessing convergence. Further calculations based on Gong and Flegal’s work [14] reveal that an ESS of 6000 measured by a 95% confidence interval corresponds to computational uncertainty of approximately 5% the size of the posterior standard deviation while the ESS recommendation of 200 made by Tracer and measured by a 95% confidence interval is equivalent to having a computational uncertainty of 28% the size of the posterior standard deviation. Therefore, the estimated variances used in calculations with Tracer’s heuristic can be unstable for the sample size of 200 and the simulation has, probably, not converged yet. Thus, we are looking forward to connect ESS based tests available in VMCMC to computational uncertainty using FWSR in the newer releases of VMCMC.

## Conclusions

VMCMC can be applied to trace files from several molecular phylogenetics MCMC tools. Assessing whether a chain has converged and is sampling from the stationary distribution is a non-trivial task. As demonstrated by the example, VMCMC can help identify issues with mixing and convergence of the MCMC run for all parameters. The graphical user interface supports interactive data exploration and the command line interface enables large-scale automated application.

VMCMC simplifies tasks in MCMC analysis that we encounter in our work and we believe that our software can be valuable to the community as well.

An executable jar file, tutorial, and source code can be downloaded from https://bitbucket.org/rhali/visualmcmc/. The tutorial is also available as Additional file 4.

## Additional files

Additional file 1 Dated species tree used in the example analysis that yielded the traces in Additional files 2 and 3. (TXT 866 KB)

Additional file 2 Sample run file generated from JPrIME. Can be opened using standard text editors or through VMCMC. (MCMC 71065.6 KB)

Additional file 3 Sample run file generated from JPrIME using the same data as Additional file 2. Can be opened using standard text editors or through VMCMC. (MCMC 71065.6 KB)

Additional file 4 Tutorial file containing all information about input, output, commands to run VMCMC from command line, GUI options, FAQ and even a list of features we have identified as interesting and which we will target for implementation in future. It is in pdf format and can be open using Adobe Acrobat Reader. (PDF 1710.08 KB)

## Abbreviations

ESS: Effective sample size
FWSR: Fixed-width stopping rule
GUI: Graphical user interface
JSON: JavaScript object notation
MCMC: Markov chain Monte Carlo
