# Supplementary material for: VMCMC: a graphical and statistical analysis tool for Markov chain Monte Carlo traces
Source: BMC Bioinformatics. 2017 Feb 10;18:97. doi: 10.1186/s12859-017-1505-3 (PMC5301390; doi:10.1186/s12859-017-1505-3)

VMCMC: a graphical and statistical analysis tool for Markov chain Monte Carlo traces in Bayesian phylogeny

Tutorial version 1.0

Last updated by **Raja Hashim Ali** on 6 Nov 2015.

Contents

1 INTRODUCTION **3**
1.1 Visual Markov Chain Monte Carlo . . . 3
1.1.1 Input . . . 3
1.1.2 Output for command line . . . 3

2 DOWNLOAD **4**

3 REQUIREMENTS **4**

4 REFERENCES **4**

5 INPUT **5**
5.1 MCMC File . . . 5

6 OUTPUT **6**

7 RUNNING **7**
7.1 Starting the application . . . 7

8 Convergence diagnostics and other analysis tools **8**
8.1 Alternate to VMCMC! . . . 8

9 OPTIONS **9**
9.1 General options . . . 9

10 GUI and command line **10**
10.1 First window . . . 10
10.2 Convergence Diagnostics . . . 10
10.3 Parameter Trace and Statistics . . . 12
10.4 Tabular Representation of Data . . . 14
10.5 Tree Parameter View and Properties . . . 14
10.6 Consensus Tree Properties . . . 15
10.7 Distance between Trees and Multi-Dimensional Scaling . . . 16
10.8 Parallel Chain Analysis for Continuous Parameters . . . 17
10.9 Parallel Chain Analysis for Tree Parameters using Splits . . . 18
10.10Sample data . . . 19

11 TIPS **20**

12 EXAMPLES **21**

13 Wish list of features for VMCMC **22**

14 FAQ **23**

15 Team **25**

1 INTRODUCTION

VMCMC has not yet been released, and the information below is up-to-date. Feedback is much appreciated, should you want to try VMCMC out. We have a stable stand-alone version uploaded on this site.

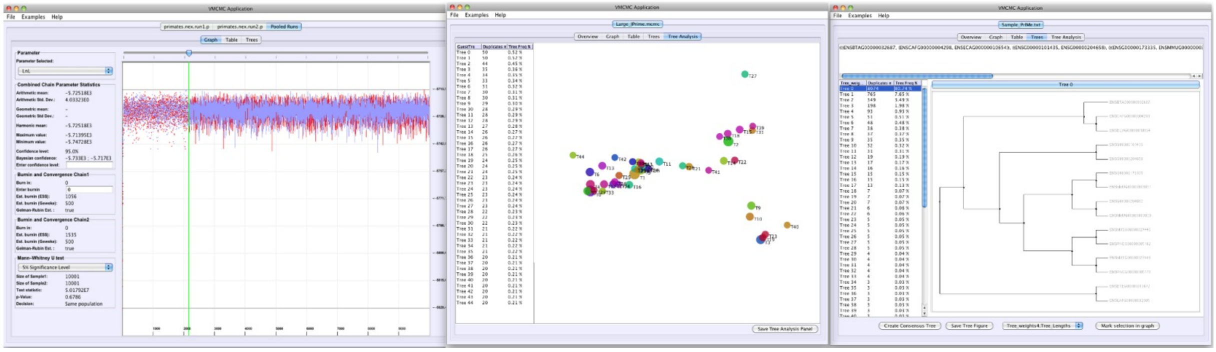

1.1 Visual Markov Chain Monte Carlo

VMCMC is an application for analyzing output of MCMC (Markov Chain Monte Carlo) chains. It deals with various metrics for assessing MCMC convergence, as well as summary statistics of inferred real-valued parameters, tree topologies, etc. VMCMC is designed for tab-delimited MCMC files but can also be used with MCMC output from the C++ version of PrIME, some applications of JPrIME (e.g., DLRS and DLTRS), MrBayes and BEAST.

VMCMC supports two modes for analyzing an MCMC chain:

1. Graphical user interface (GUI) for showing trace plots, etc.
2. Command-line summary of important statistics.

Hence the name VMCMC = Visual Markov Chain Monte Carlo.

VMCMC is a Java application that entails many computational algorithms and statistical techniques to analyze MCMC chains statistically and visually. The command line output can be parsed using any JSON parser and therefore VMCMC has the ability to be automated (thereby can be used as part of pipeline).

1.1.1 Input

Nothing or alternatively a MCMC file.

1.1.2 Output for command line

JSON formatted file with inquired statistics. Below, you will find information on the input and output of the application, while explanations of vital program options and some sample files can be found at the end of the document. If you want to get started quickly, we suggest that you download and try Example 1, and have a look at it while reading through the other parts of the guide.

2 DOWNLOAD

VMCMC is distributed as a Java executable and is included in the VMCMC JAR file, whose up-to-date version can be obtained from here.

Older VMCMC executables can be found here.

3 REQUIREMENTS

VMCMC requires Java SE 6 but can also run on later versions. However, on version 7 (at least), the 2D plotting functionality is slower than on version 6 and therefore, the Graph panel takes a lot of time to load and significant delay is observed. Hence, Java SE 6 is recommended for VMCMC.

4 REFERENCES

VMCMC is not yet accepted or published but we expect it to be published in some reputed journal soon. If you use it, please look at this site for updates on citation.

5 INPUT

5.1 MCMC File

The MCMC file should be a tab delimited file containing samples consisting of one or more parameters. Additionally, it can be computed with any suitable Bayesian phylogeny inference program (PrIMe, JPrIMe, BEAST or MrBayes currently) and should be provided without any editing e.g. following is a snapshot of an output from JPrIMe and the complete example can be followed in Example 1:

```
Iteration	OverallLikelihood	SubstitutionModelLikelihood	DLRSModelLikelihood	DuplicationRate
LossRate	EdgeRateMean	EdgeRateCV	GuestTree
0	-2781.029156	-2688.241825	-92.78733099	0.846717234	-0.846717234	0.5	0.5	-
((Cavia_porcellus_1,(((Equus_caballus_1,Monodelphis_domestica_1),Oryctolagus_cuniculus_1),Felis_catus_1)),Mus_musculus_1);
100	-2598.675621	-2572.196059	-26.47956158	0.201412555	1.470475779	0.46883284	0.708159733	-
((((Cavia_porcellus_1,Mus_musculus_1),Felis_catus_1),(Monodelphis_domestica_1,Oryctolagus_cuniculus_1)),Equus_caballus_1);
200	-2579.528545	-2550.919071	-28.60947335	0.285730641	2.360374081	0.628144043	1.330175626	-
((((Cavia_porcellus_1,Mus_musculus_1),Felis_catus_1),(Monodelphis_domestica_1,Oryctolagus_cuniculus_1)),Equus_caballus_1);
300	-2577.054947	-2548.483723	-28.57122338	1.080013459	1.060243314	0.478689404	1.313385769	-
(((((Cavia_porcellus_1,Mus_musculus_1),Felis_catus_1),Monodelphis_domestica_1),Oryctolagus_cuniculus_1),Equus_caballus_1);
400	-2561.411298	-2541.905901	-19.50539698	2.335910787	1.709855994	0.621885156	1.062674522	-
(((Cavia_porcellus_1,Mus_musculus_1),Oryctolagus_cuniculus_1),((Equus_caballus_1,Felis_catus_1),Monodelphis_domestica_1));
500	-2553.419283	-2539.158567	-14.26071523	0.743094603	0.788164172	0.44922322	1.190562139	-
((((Cavia_porcellus_1,Mus_musculus_1),Oryctolagus_cuniculus_1),Equus_caballus_1),(Felis_catus_1,Monodelphis_domestica_1));
600	-2554.743447	-2539.582612	-15.16083501	0.772467743	1.90347028	0.488871609	1.000954121	-
((((Cavia_porcellus_1,Mus_musculus_1),Oryctolagus_cuniculus_1),Equus_caballus_1),(Felis_catus_1,Monodelphis_domestica_1));
700	-2551.984337	-2537.760924	-14.22341271	0.406903538	0.564372111	0.301825965	1.060518568	-
((((Cavia_porcellus_1,Mus_musculus_1),Oryctolagus_cuniculus_1),Equus_caballus_1),(Felis_catus_1,Monodelphis_domestica_1));
800	-2554.544787	-2539.398353	-15.14643407	0.705793718	2.283847179	0.390386464	0.832413684	-
((((Cavia_porcellus_1,Mus_musculus_1),Oryctolagus_cuniculus_1),Equus_caballus_1),(Felis_catus_1,Monodelphis_domestica_1));
900	-2554.79669	-2540.458545	-14.33814436	1.429817872	1.668677683	0.366982526	1.061218512	-
((((Cavia_porcellus_1,Mus_musculus_1),Oryctolagus_cuniculus_1),Equus_caballus_1),(Felis_catus_1,Monodelphis_domestica_1));
1000	-2554.725103	-2540.934668	-13.79043577	0.920909518	1.803576387	0.333953057	0.869808796	-
(((((Cavia_porcellus_1,Mus_musculus_1),Oryctolagus_cuniculus_1),Felis_catus_1),Monodelphis_domestica_1),Equus_caballus_1);
...
```

Note that VMCMC expects standard MCMC chain output from these software. If the numeric parameters in these software contain any String values or the tree provided is not in Newick or Nexus format or the output from these software is edited (except for sample deletion or whole line deletions from data) in particular the header line is removed, then VMCMC will give an error. So please make sure that the input to VMCMC is exactly as output by the originating software.

6 OUTPUT

Output is typically shown in GUI. However, with proper options, it can also be directed to a file in JSON format and a JSON parser can be employed to extract and understand the output. Following is the output of the posterior tree distribution through command line using the option ”-p”.The file with the samples will look something like:


```
{
        "File": "/Users/rhali/Documents/Eclipse/workspace/VMCMC/src/main/resources/Sample_JPrIMe.mcmc",
        "Total_iterations": 10001,
        "Burnin": 2551,
        "Trees": {
                "Series_0": [
                        {
                                "Index": 0,
                                "Duplicates": 7406,
                                "Posterior probability": 0.99,
                                "Newick":
"((((Cavia_porcellus_1,Mus_musculus_1),Oryctolagus_cuniculus_1),(Equus_caballus_1,Felis_catus_1)),Monodelphis_domestica_1);"
                        },
                        {
                                "Index": 1,
                                "Duplicates": 28,
                                "Posterior probability": 0.00,
                                "Newick":
"(((((Cavia_porcellus_1,Mus_musculus_1),Oryctolagus_cuniculus_1),Felis_catus_1),Equus_caballus_1),Monodelphis_domestica_1);"
                        },
                        {
                                "Index": 2,
                                "Duplicates": 16,
                                "Posterior probability": 0.00,
                                "Newick":
"(((((Cavia_porcellus_1,Mus_musculus_1),Oryctolagus_cuniculus_1),Equus_caballus_1),Felis_catus_1),Monodelphis_domestica_1);"
                        }
                ]
        }
}
```

7 RUNNING

7.1 Starting the application

VMCMC can be started as a command-line Java application as well as with graphical user interface. You would start VMCMC graphical user interface by running e.g.:

```
java -jar VMCMC-X.Y.Z.jar <filename>
```

or preferably without filename.

```
java -jar VMCMC-X.Y.Z.jar
```

or for command line output, use the corresponding option.

```
java -jar VMCMC-X.Y.Z.jar [option(s)] <filename>
```

where the JAR file of course refer to your current setup. See also the section on options below. You may need to increase the default heap size allocated by Java in case of very large MCMC files usually in excess of 20k samples. The memory requirements will primarily depend on the size of your MCMC data. We recommend using Oracle's Java HotSpot? virtual machine (VM), in which case a recommended heap size of 512 MB and a maximum heap size of 1024 MB would be specified with:

```
java -Xms512m -Xmx1024m -jar VMCMC-X.Y.Z.jar [options] <filename>
```

Note that these options refer to Java's VM itself, and are not options of VMCMC. Tuning other aspects of a Java VM is a non-trivial area and is probably not necessary. Should you still wish to do so, please have a look at these documents: Oracle Java HotSpot VM FAQand Oracle Java HotSpot VM Options.

8 Convergence diagnostics and other analysis tools

Several MCMC-tailored analysis softwares exist, among them:

- The CRAN R package CODA.
- Tracer.
- AWTY. Particularly tailored for inspecting clade convergence diagnostics.

8.1 Alternate to VMCMC!

Generally, CRAN R is suitable for many types of analyses. For instance, you could read and plot the duplication rate in R thus:

```
> burnInLim <- 250000;
> chain <- read.table('myoutput.mcmc', header=TRUE);
> chain <- chain[chain$Iteration > burnInLim, ];
> hist(chain$DuplicationRate, 40);
```

To get a quick glimpse of the tree space, you could even use a shell such as bash (assuming the tree is in column 9 and we only look at the last 5,000 samples):

```
$ cut -f9 myoutput.mcmc | tail -5000 | sort | uniq -c | sort -n
```

This will quickly identify the maximum probability tree among the 5,000 samples.
But rest assured, VMCMC is much easier to use with a lot more functionality and much more deterministic e.g. for convergence diagnostics and tree parameters.

9 OPTIONS

VMCMC comes with a large number of user options; please don't feel overwhelmed! All options are detailed below. You can always type

```
java -jar VMCMC-X.Y.Z.jar -h
```

to get a more up-to-date but brief description of all available options. Notice that some options have precedence over others and will display the highest prioritized option of the selected ones only.

9.1 General options

- -n Test and simple statistics shown on command line only. VMCMC computed statistics and convergence test results and/or burnin estimate for each parameter shown on stdout. By default is turned off.
- -c<int> Confidence Level value e.g. 90. Default: 95.
- -t Test statistics shown on command line only. VMCMC computed test results for each parameter shown on stdout. By default is turned off.
- -s Statistics shown on command line only. VMCMC computed statistics for the MCMC chain shown on stdout. By default is turned off.
- -g Geweke convergence test and burn in estimator result for each parameter on command line only. VMCMC computed Geweke convergence and burn in estimator for the MCMC chain shown on stdout. It does not set the burnin to Geweke estimated burnin for other options but will estimate and output the burnin on command line. By default is turned off.
- -e Effective Sample Size test result on command line only. VMCMC computed estimated sample size burn-in and convergence estimator for the MCMC chain shown on stdout. It does not set the burnin to ESS estimated burnin for other options but will estimate and output the burnin on command line. By default is turned off.
- -r Gelman-Rubin convergence test result on command line only. VMCMC computed gelman rubin burn-in and convergence estimator for the MCMC chain shown on stdout. It does not set the burnin to Gelman-Rubin estimated burnin for other options but will estimate and output the burnin on command line. By default is turned off.
- -p Display Posterior distribution of trees. By default is turned off.
- -b<int> Burn-in samples to remove from trace for command line analysis for single chains only. By default is -1.
- -b1<int> Burn-in samples to remove from trace for command line analysis for first chain among parallel chains. By default is -1.
- -b2<int> Burn-in samples to remove from trace for command line analysis for second chain among parallel chains. By default is -1.
- -m Calculate and display MAP tree. By default is turned off.
- -ct Run the global convergence tests only. By default is turned off.
- -sd Sample a data point uniformly from converged MCMC chain. By default is turned off.
- -o Output command line options on standard output or in a specified file. By default is set to stdout.
- -pa Path where to make the output file for command line VMCMC. Default: ./.
- -a<float> Alpha value/Level of significance for parallel chain analysis. Default: 0.05.

10 GUI and command line

10.1 First window

If VMCMC is ran without any arguments, then the first Graphical User Interface appears with **File** menu. The user can choose if he wants to open example runs from various software from **Examples** menu or if they want to open a file from the hard disk. Furthermore, it contains the help menu referring to this website tutorial and also the names of members of the team responsible for VMCMC in the **About** menu.

From the command line, the first window can be accessed by the following command.

```
$ java -Xmx2000m -Xms1800m -jar VMCMC-X.Y.Z.jar
```

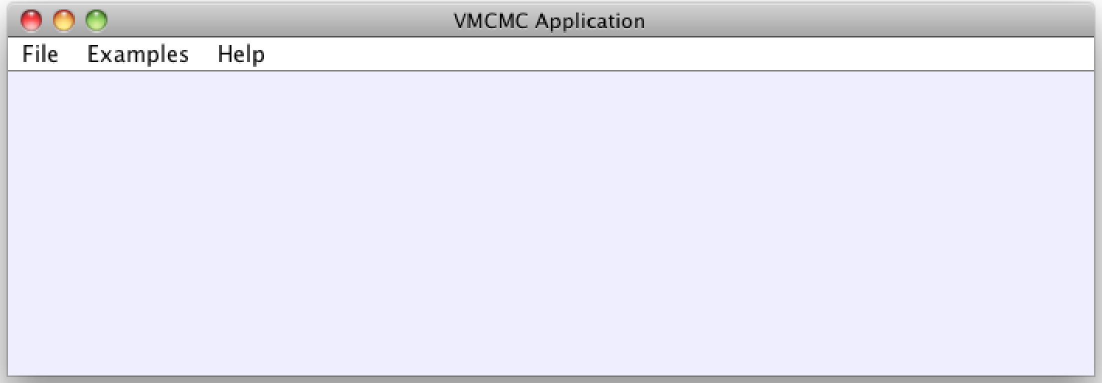

10.2 Convergence Diagnostics

VMCMC has several established parameter convergence diagnostics like Geweke and Gelman Rubin. These are displayed on the right panel for each parameter. While Geweke follow the traditional definitions, Gelman Rubin parameter burnin estimates are defined by "the first sample value for which gelman rubin diagnostic results as converged for a parameter".

From the command line, the Geweke convergence diagnostic for each parameter can be calculated by the following command.

```
$ java -Xmx2000m -Xms1800m -jar VMCMC-X.Y.Z.jar <MCMC File> -g -o <Output>
```

From the command line, the Gelman Rubin convergence diagnostic for each parameter can be calculated by the following command.

```
$ java -Xmx2000m -Xms1800m -jar VMCMC-X.Y.Z.jar <MCMC File> -r -o <Output>
```

The $\mathrm{ESS}_{\mathrm{Max}}$ or Sahlin-Höhna ESS Burnin estimator ($SHEB$) is however not so commonly used. Please note that it does not display the ESS value for a given burnin but determines the sample number at which the ESS value is maximized for a given parameter chain. Sebastian Höhna and Kristoffer Sahlin should be credited for proposing this diagnostic for a single parameter. It will be interesting to show the actual ESS value observed for this burnin as well in a separate column for making convergence assessment decision easy (e.g., by using the Tracer heuristic of greater than 200 for converged and for less than 100 as not converged). Please refer to Kristoffer Sahlin Master thesis titled "Estimating convergence of Markov chain Monte Carlo simulations" for theory and estimation method for $\mathrm{ESS}_{\mathrm{Max}}$ (exact formula on page 15).

From the command line, the ESS_{Max} convergence diagnostic for each parameter can be calculated by the following command.

```
$ java -Xmx2000m -Xms1800m -jar VMCMC-X.Y.Z.jar <MCMC File> -e -o <Output>
```

From the command line, the convergence assessment and burnin estimates of all three convergence diagnostics for each parameter can be calculated by the following command.

```
$ java -Xmx2000m -Xms1800m -jar VMCMC-X.Y.Z.jar <MCMC File> -t -o <Output>
```

Of particular interest are the **global convergence diagnostics** which are not available or have not been established yet. What is the overall convergence status of the whole MCMC chain and not of the individual parameters? If the chain has converged, what is the universal burnin for all numeric parameters? VMCMC attempts to answer these questions using the global convergence diagnostics and we are in the process of establishing these burnin estimation and convergence assessment diagnostics for the complete run including all parameters. A basic study that establishes why these diagnostics are important will be coming soon in the near future! The global convergence diagnostics are shown in the left panel below.

1. $\text{ESS}_{\text{Last Burnin}}$ is the maximum of all parameter estimates for ESS_{Max} or Sahlin-Höhna ESS Burnin estimator ($SHEB$). We have used the maximum of all parameter burnins concept using this estimator to estimate a universal burnin for multiple parameters.
2. $\text{Geweke}_{\text{Last Burnin}}$ is the maximum of all parameter estimates for Geweke and not converged if any parameter in the chain has not converged according to Geweke convergence diagnostic.
3. Gelman-Rubin_{LB} is the maximum of all parameter estimates for Gelman-Rubin and not converged if any parameter in the chain has not converged according to Gelman-Rubin convergence diagnostic.
4. Standaradized ESS standardizes all numeric parameters and then estimates convergence for the whole vector per sample (treating it as a n-dimensional data with n = number of parameters) instead of a single point per sample. This removes the bias for each parameter and brings the scale to the same level.
5. Normalized ESS normalizes all numeric parameters and then estimates convergence for the whole vector per sample (treating it as a n-dimensional data with n = number of parameters) instead of a single point per sample. This removes the bias for each parameter and brings the scale to between 0 and 1 for all parameters.

From the command line, the global convergence diagnostics can be applied by the following command and the burnin and convergence can be assessed for the complete chain.

```
$ java -Xmx2000m -Xms1800m -jar VMCMC-X.Y.Z.jar <MCMC File> -ct -o <Output>
```

Please note that by default, the burnin suggested by $ESS_{LastBurnin}$ is selected as the burnin for all parameters in the chain for the GUI-based VMCMC and from the command line based VMCMC (if no burnin has been supplied) for those analysis that require burnin.

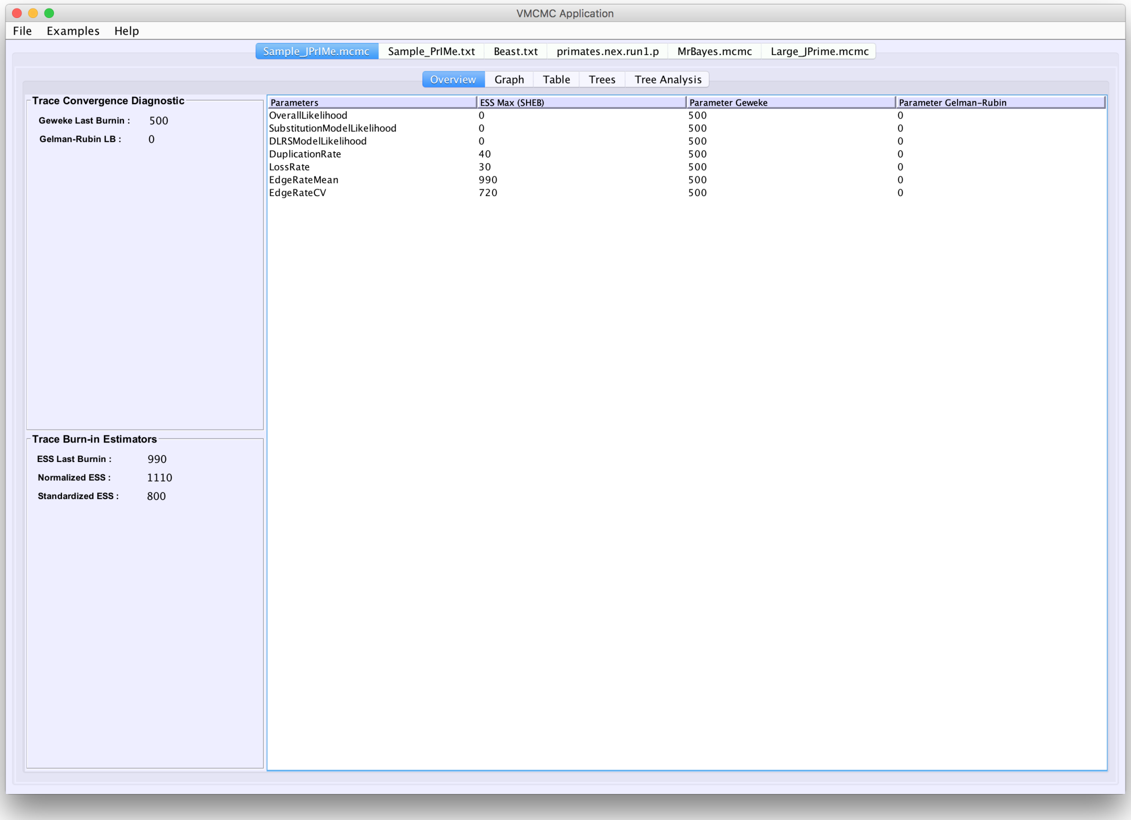

10.3 Parameter Trace and Statistics

MCMC trace is another important characteristic of MCMC chains and trace of selected parameter from the dropdown menu is shown graphically on the right hand side of the tab. Mixing of MCMC for numeric parameters is seen and visual estimation of convergence and burnin can be performed using the parameter trace.

Parameter statistics for the selected parameter are shown on the left hand side with minimum and maximum values, arithmetic, harmonic and geometric means, arithmetic and geometric standard deviations, Bayesian confidence interval and burnin and convergence estimates are shown. These values can be used to verify the mixing of each parameter numerically and in case of command-line automate this verification of mixing as well as accuracy of MCMC chains (if true parameter value, if known, exists within 95% confidence interval).

From the command line, the parameter statistics can be determined by the following command with the specified burnin or with the burnin estimated from $ESS_{LastBurnin}$ if burnin is not specified and determine the credible interval using the specified confidence level.

```
$ java −Xmx2000m −Xms1800m −jar VMCMC−X.Y.Z.jar <MCMC File> −s
−o <Output> −b <Burnin> −c <ConfidenceLevel>
```

An important characteristic of VMCMC is the ability to extract a selected interval and display it in a new tab (which is like zooming in on a particular window and estimating its statistics and visualizing its trace closely).

Another option available from command line is to determine the convergence test results and parameter statistics by the following command.

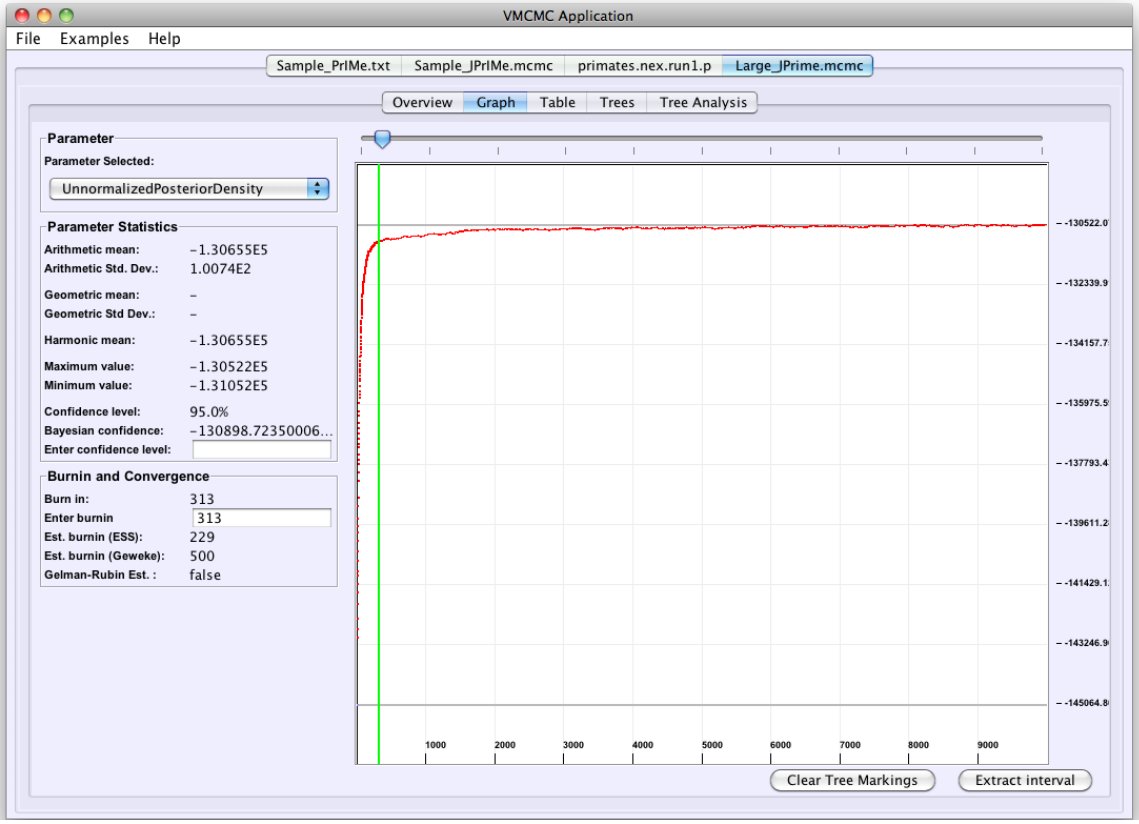

$ java −Xmx2000m −Xms1800m −jar VMCMC−X.Y.Z.jar <MCMC File> −n −o <Output>

10.4 Tabular Representation of Data

Numeric parameters are presented in tabular format, so that values of each parameters are easily readable and user can see the data in a good (Microsoft excel-like) presentation.

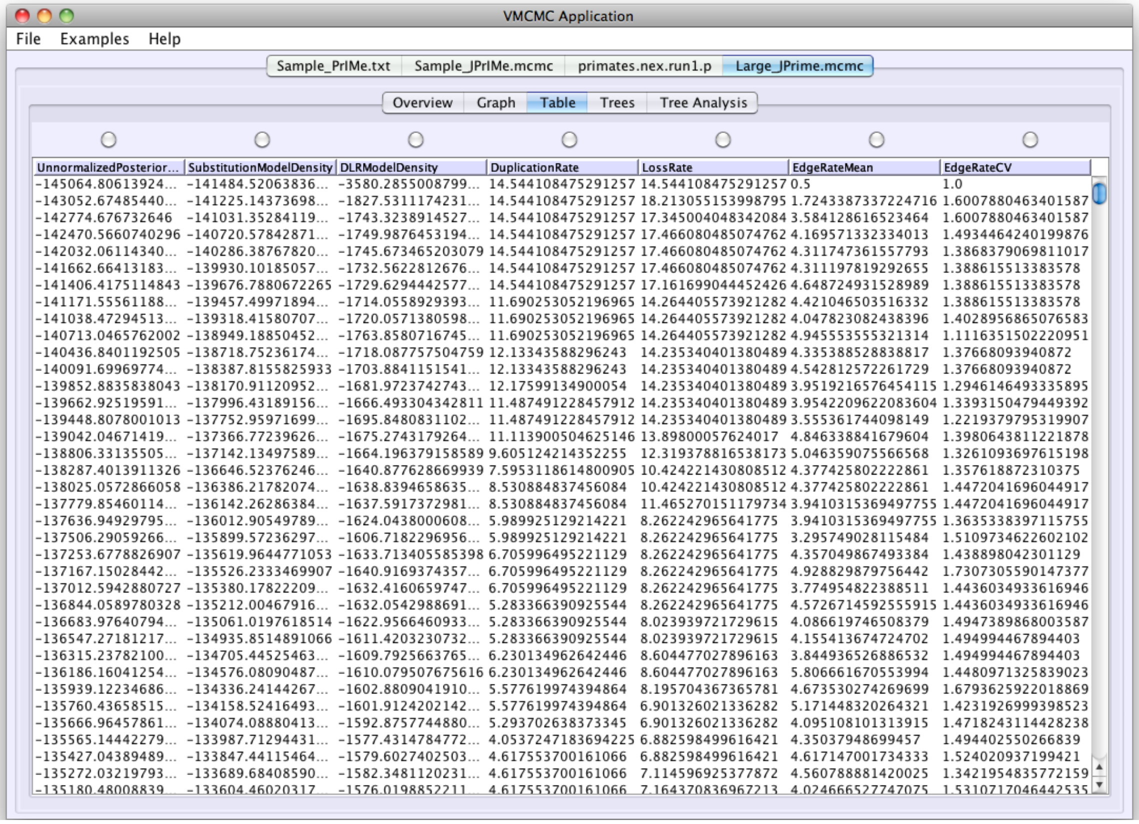

UnnormalizedPosterior...	SubstitutionModelDensity	DLRModelDensity	DuplicationRate	LossRate	EdgeRateMean	EdgeRateCV
-145064.80613924...	-141484.52063836...	-3580.2855008799...	14.544108475291257	14.544108475291257	0.5	1.0
-143052.67485440...	-141225.14373698...	-1827.5311174231...	14.544108475291257	18.213055153998795	1.7243387337224716	1.6007880463401587
-142774.676732646	-141031.35284119...	-1743.3238914527...	14.544108475291257	17.345004048342084	3.584128616523464	1.6007880463401587
-142470.5660740296	-140720.57842871...	-1749.9876453194...	14.544108475291257	17.466080485074762	4.169571332334013	1.4934464240199876
-142032.06114340...	-140286.38767820...	-1745.673465203079	14.544108475291257	17.466080485074762	4.311747361557793	1.3868379069811017
-141662.66413183...	-139930.10185057...	-1732.5622812676...	14.544108475291257	17.466080485074762	4.311197819292655	1.388615513383578
-141406.4175114843	-139676.7880672265	-1729.6294442577...	14.544108475291257	17.161699044452426	4.648724931528989	1.388615513383578
-141171.55561188...	-139457.49971894...	-1714.0558929393...	11.690253052196965	14.264405573921282	4.421046503516332	1.388615513383578
-141038.47294513...	-139318.41580707...	-1720.0571380598...	11.690253052196965	14.264405573921282	4.047823082438396	1.4028956865076583
-140713.0465762002	-138949.18850452...	-1763.8580716745...	11.690253052196965	14.264405573921282	4.945553555321314	1.1116351502220951
-140436.8401192505	-138718.75236174...	-1718.087757504759	12.13343588296243	14.235340401380489	4.335388528838817	1.37668093940872
-140091.69969774...	-138387.8155825933	-1703.8841151541...	12.13343588296243	14.235340401380489	4.542812572261729	1.37668093940872
-139852.8835838043	-138170.91120952...	-1681.9723742743...	12.17599134900054	14.235340401380489	3.9519216576454115	1.2946146493335895
-139662.92519591...	-137996.43189156...	-1666.493304342811	11.487491228457912	14.235340401380489	3.9542209622083604	1.3393150479449392
-139448.8078001013	-137752.95971699...	-1695.8480831102...	11.487491228457912	14.235340401380489	3.555361744098149	1.2219379795319907
-139042.04671419...	-137366.77239626...	-1675.2743179264...	11.113900504625146	13.89800057624017	4.846338841679604	1.3980643811221878
-138806.33135505...	-137142.13497589...	-1664.196379158589	9.605124214352255	12.319378816538173	5.046359075566568	1.3261093697615198
-138287.4013911326	-136646.52376246...	-1640.877628669939	7.5953118614800905	10.424221430808512	4.377425802222861	1.357618872310375
-138025.0572866058	-136386.21782074...	-1638.8394658635...	8.530884837456084	10.424221430808512	4.377425802222861	1.4472041696044917
-137779.85460114...	-136142.26286384...	-1637.5917372981...	8.530884837456084	11.465270151179734	3.9410315369497755	1.4472041696044917
-137636.94929795...	-136012.90549789...	-1624.0438000608...	5.989925129214221	8.262242965641775	3.9410315369497755	1.3635338397115755
-137506.29059266...	-135899.57236297...	-1606.7182296956...	5.989925129214221	8.262242965641775	3.295749028115484	1.5109734622602102
-137253.6778826907	-135619.9644771053	-1633.713405585398	6.705996495221129	8.262242965641775	4.357049867493384	1.438898042301129
-137167.15028442...	-135526.2333469907	-1640.9169374357...	6.705996495221129	8.262242965641775	4.928829879756442	1.7307305590147377
-137012.5942880727	-135380.17822209...	-1632.4160659747...	6.705996495221129	8.262242965641775	3.77495482238851	1.4436034933616946
-136844.0589780328	-135212.00467916...	-1632.0542988691...	5.283366390925544	8.262242965641775	4.5726714592555915	1.4436034933616946
-136683.97640794...	-135061.0197618514	-1622.9566460933...	5.283366390925544	8.023939721729615	4.086619746508379	1.4947389868003587
-136547.27181217...	-134935.8514891066	-1611.4203230732...	5.283366390925544	8.023939721729615	4.155413674724702	1.494994467894403
-136315.23782100...	-134705.44525463...	-1609.7925663765...	6.230134962642446	8.604477027896163	3.844936526886532	1.494994467894403
-136186.16041254...	-134576.08090487...	-1610.079507675616	6.230134962642446	8.604477027896163	5.806661670553994	1.4480971325839023
-135939.12234686...	-134336.24144267...	-1602.8809041910...	5.577619974394864	8.195704367365781	4.673530274269699	1.6793625922018869
-135760.43658515...	-134158.52416493...	-1601.9124202142...	5.577619974394864	6.901326021336282	5.171448320264321	1.4231926999398523
-135666.96457861...	-134074.08880413...	-1592.8757744880...	5.293702638373345	6.901326021336282	4.095108101313915	1.4718243114428238
-135565.14442279...	-133987.71294431...	-1577.4314784772...	4.0537247183694225	6.882598499616421	4.35037948699457	1.494402550266839
-135427.04389489...	-133847.44115464...	-1579.6027402503...	4.617553700161066	6.882598499616421	4.617147001734333	1.524020937199421
-135272.03219793...	-133689.68408590...	-1582.3481120231...	4.617553700161066	7.114596925377872	4.560788881420025	1.3421954835772159
-135180.48008839...	-133604.46020317...	-1576.0198852211...	4.617553700161066	7.164370836967213	4.024666527747075	1.5310717046442535

10.5 Tree Parameter View and Properties

Tree parameter is an important parameter of MCMC analysis. Estimating the true posterior distribution and searching for MAP tree is a difficult task and in particular determining equivalent trees with different Newick and Nexus representation is not possible using conventional cut, tail, sort and uniq method. Therefore first the branch lengths of trees are removed and then we estimate the posterior distribution for trees. The output is sorted and displayed with tree numberings, number of trees with this topology and frequency within the chain after removing burnin samples (determined by ESSMax) on the left hand panel. At the top are the newick string(s) of the selected tree(s). At the right hand panel, the selected tree(s) are shown in cladogram(s) using forester library.

From the command line, the tree posterior can be determined by the following command with the specified burnin or with the burnin estimated from $ESS_{LastBurnin}$ if burnin is not specified.

```
$ java -Xmx2000m -Xms1800m -jar VMCMC-X.Y.Z.jar <MCMC File> -p
-o <Output> -b <Burnin>
```

From the command line, the maximum *a posteriori* tree can be determined by the following command with the specified burnin or with the burnin estimated from $ESS_{LastBurnin}$ if burnin is not specified.

```
$ java -Xmx2000m -Xms1800m -jar VMCMC-X.Y.Z.jar <MCMC File> -m
-o <Output> -b <Burnin>
```

An important characteristic of VMCMC is its ability to display how a numeric parameter behaves for a selected tree from the tree posterior, i.e., what were the parameter statistics for a selected numeric parameter (selected from drop-down menu) for a selected tree in the tree panel. Therefore selecting tree(s) from left hand side and pressing **Mark Selection in Graph** button will highlight the corresponding regions in parameter trace in Graph tab. Switching to Graph tab, one can visualize the ares, this tree topology is observed and **Clear Tree Markings** will remove these marks. Notice that each tree selected has a unique color although at this moment, there is no mapping available for a color to a tree.

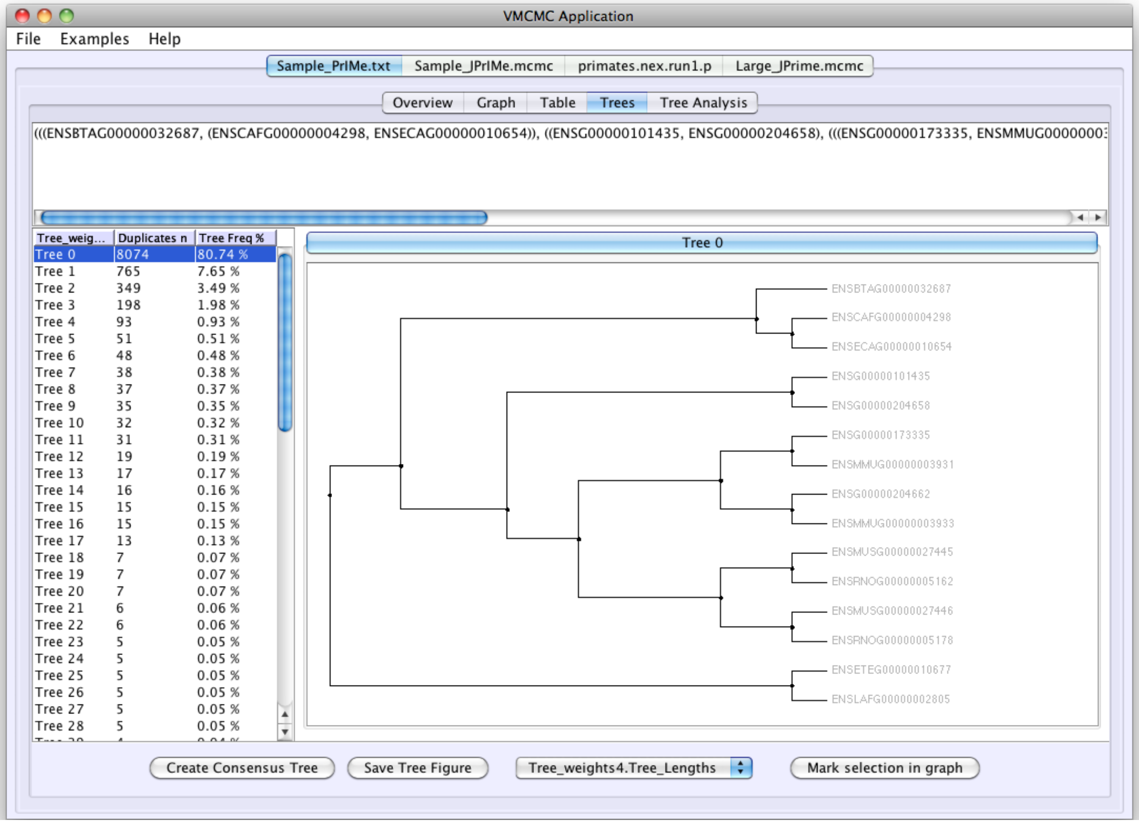

10.6 Consensus Tree Properties

Another interesting property of the trees is to see which trees are closer to each other and which are not. This can be done in two ways, through the consensus tree which shows the maximum splits based on frequency of selected trees and secondly through the Multi-Dimensional Scaling (MDS) technique discussed later. The consensus tree can be determined for all trees if one or none of the trees is selected. If two or more trees are selected, then the consensus tree for these trees will be computed based on the majority splits on tree frequency.

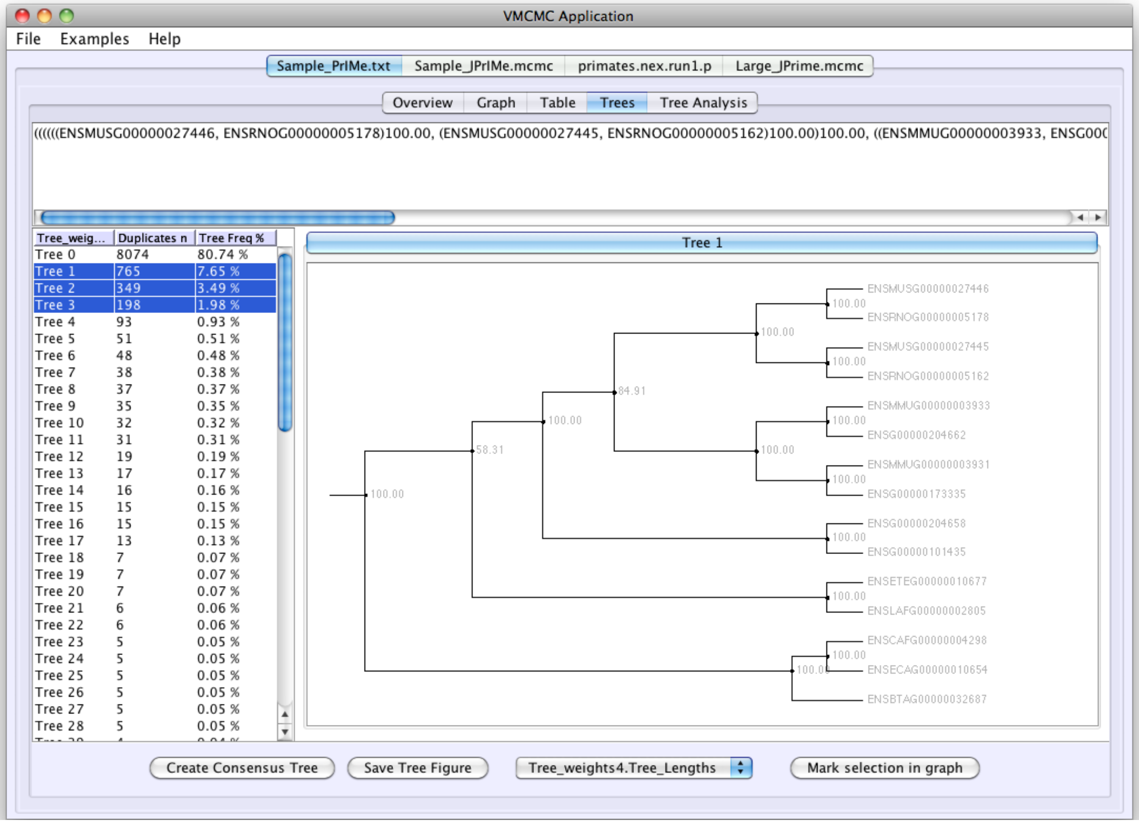

10.7 Distance between Trees and Multi-Dimensional Scaling

The second way to estimate distance between all trees is Multi-Dimensional Scaling (MDS) technique which displays the tree posterior in 2 Dimensional space as an edgeless graph, where each tree is represented by a labelled vertex (whose size is proportional to tree frequency and whose label is T + the tree number). First a 2Dimensional distance matrix is constructed, which takes as input all tree topologies with frequency greater than 0.2% and Robinson Fould's distances between two trees are computed and stored in the matrix. Then this matrix is passed to MDS scaling module, which fits these trees onto two dimensions according to the scaled distance between the trees and minimizing the difference (error).

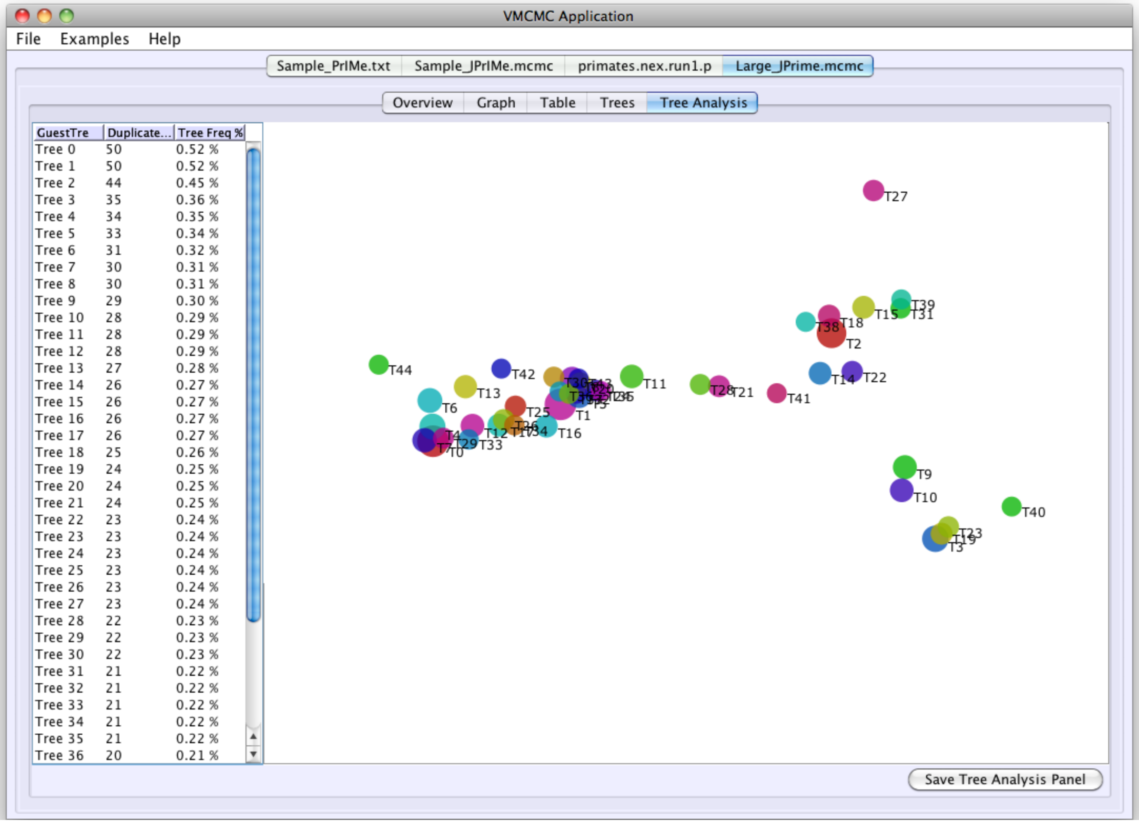

10.8 Parallel Chain Analysis for Continuous Parameters

If two chains have ran in parallel with the same data and using the same software, how can we determine, if the samples have been sampled from the same posterior distribution? More importantly, if so, then how can we combine this information and get maximum usage of parallel chains? VMCMC addresses this question in three steps. The first way is that Mann Whitney U test is applied for numeric parameters for a given level of significance and decision for this parameter is shown at the bottom left side of the parallel chain trace window. The second step is pooling of tree parameters after MCMC convergence and using this data to infer tree related properties like tree posterior distribution and tree analysis tab etc. Please note that after removing the specified/default burnin samples from both traces, remaining samples in both chains are combined and statistics for a combined trace are displayed.

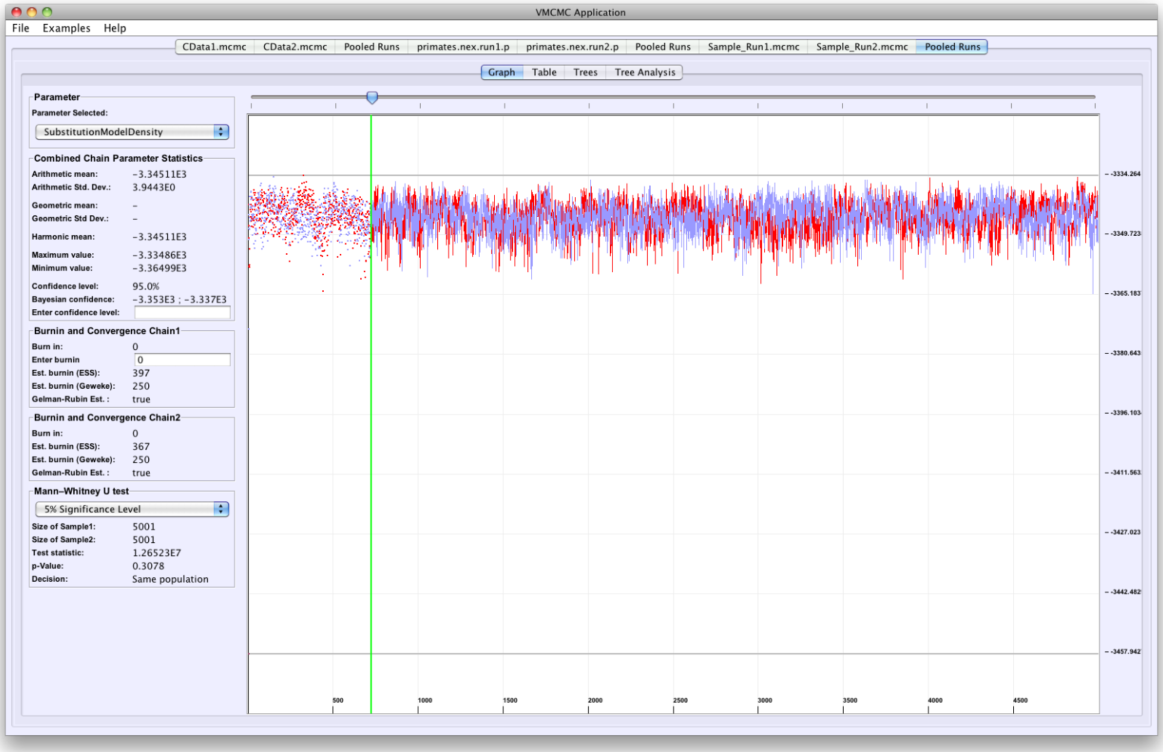

10.9 Parallel Chain Analysis for Tree Parameters using Splits

VMCMC uses tree splits to identify if both the tree parameter posterior distribution have been sampled from the same distribution using the Chi square for two independent samples test for a given level of significance. We acknowledge MrBayes for this measure. Burnin value are taken from trace window and after removing the samples in burnin period, the tree split bins are computed on which Chi square test is applied and the Chi Square test statistic, p-value and acceptance/rejection of null hypothesis are displayed on the left hand side. Please note that after removing the specified/default burnin samples from both traces, the remaining trees in the posterior of both chains are combined and displayed for the combined trace. The Mann Whitney U test for continuous parameters and Chi Square test for tree splits can also be applied through command-line with pre-specified burnin for both chains or with the burnin calculated from $ESS_{Lastburnin}$ for both chains with a specified alpha value.

```
$ java -Xmx2000m -Xms1800m -jar VMCMC-X.Y.Z.jar <MCMC Run 1> <MCMC Run 2>
-o <Output> -b1 <Burnin1> -b2 <Burnin2> -a <alpha>
```

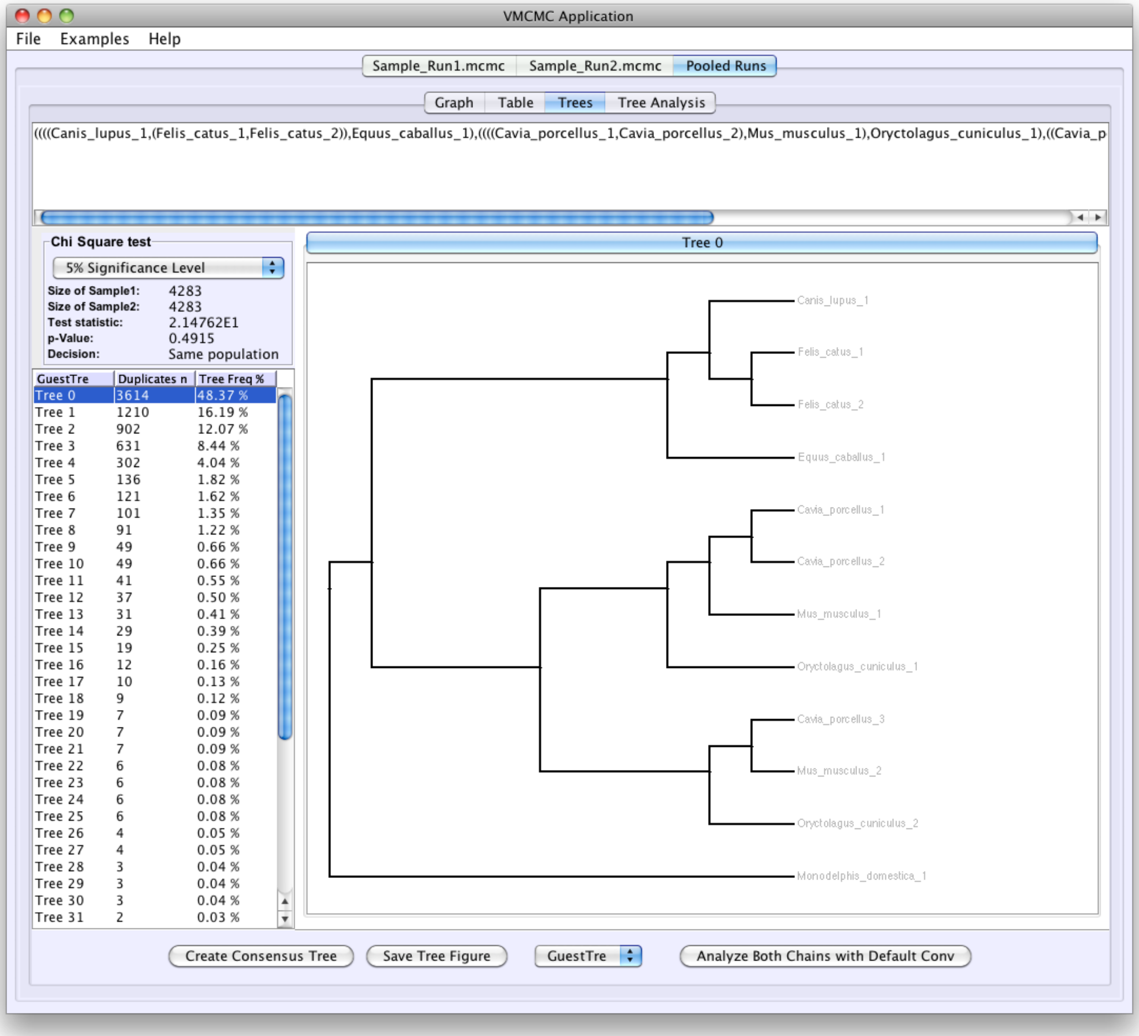

10.10 Sample data

This option is only available from command line. If one wants to randomly select a single sample from converged part of the chain, then the following command can be used with the specified burnin or with the burnin estimated from $ESS_{LastBurnin}$ if burnin is not specified.

```
$ java −Xmx2000m −Xms1800m −jar VMCMC−X.Y.Z.jar <MCMC File> −sd −b <Burnin>
−o <Output>
```

11 TIPS

VMCMC is a simple application with single input even for parallel analysis. Following are a few tips in order to use GUI for single chain and parallel chain analyses.

1. **Single chain analysis:** The input file for single chain analyses is a single MCMC file for CODA, PrIMe and JPrIMe and two files (with same names but with different extensions where one ends with .p and other with .t) for MrBayes?. For GUI, first run the program without providing any arguments. Then click the File menu on the first GUI and click the "Open File" submenu. Select the MCMC file for JPrIMe, PrIMe and CODA and the file ending with .p for MrBayes. Note that "File" menu does not contain any "Parallel Analysis" submenu. This is hidden and will appear once a file has been opened using "Open File" submenu.

2. **Parallel chain analyses:** Parallel chain analyses is performed on two chains for the **same program** ran on the **same data**. Furthermore at this point of time, the number of samples in both chains must be **same** because the trace window is unable to handle empty data for one chain at this point of time. The Chi-Square test and Mann-Whitney U-test as well as other algorithms and testing utilities are however not effected by this limitation.

Go to File → Parallel Chain Analysis and select the second file under the same restrictions for Single chain analyses. For JPrIMe, CODA and PrIMe files, select the chain with which you want to perform Parallel Chain analysis of the first chain. For MrBayes?, select the file with extension ".p" of the chain with which you want to perform Parallel Chain analysis of the first chain. Note that parallel analysis can only be performed if a single file has been opened using the "Open File" submenu otherwise this submenu will not appear on "File" menu. Furthermore, before this analysis, "File" menu contained "Parallel Analysis" submenu. This is now hidden and will not appear once a parallel analysis has been performed. To perform another parallel analysis, open a file using "Open File" submenu and it will be possible to perform a parallel analysis with this opened chain.

12 EXAMPLES

All these examples are already in the application itself. When one runs VMCMC, they can be found under the Examples submenu. However, the original data files are not visible in the application examples and so here we give the example files for better understanding of the input and functionality of VMCMC. Read Useful Tips and GUI in line with these example files to run and understand the output and functionality provided by VMCMC.

1. **JPrIMe Single chain Example**

2. **PrIMe Single chain Example**

3. **MrBayes Single chain Example**

   Download File 1 and File 2 and open primates.nex.run1.p file to see trace and other properties. MrBayes generates two files per MCMC chain, one containing the numeric parameters (ending with .p) and the other containing tree parameters (ending with .t). VMCMC assumes that both files are in the same folder.

4. **JPrIMe Single chain with large number of samples Example**

5. **MrBayes Parallel chains Example**

   Download File 1, File 2, File 3 and File 4 and open primates.nex.run1.p file using file open menu to see first chain and then open primates.nex.run2.p using Parallel file opener (hidden before but appears after one file has been opened using file open submenu) to see both chains in same window as well as separately.

6. **JPrIMe Parallel chains Example** Download File 1 and File 2 and open Sample_Run1.mcmc file using file open menu to see first chain and then open Sample_Run2.mcmc using Parallel file opener (hidden before but appears after one file has been opened using file open submenu) to see both chains in same window as well as separately.

7. **PrIMe Parallel chains Example**

   Download File 1 and File 2 and open CData1.mcmc file using file open menu to see first chain and then open CData2.mcmc using Parallel file opener (hidden before but appears after one file has been opened using file open submenu) to see both chains in same window as well as separately.

13 Wish list of features for VMCMC

Following is a list of features that we, the authors and coders have identified as interesting and which we plan to implement in near future. You are most welcome to email any of the developers with suggestions or features that you feel are interesting for your applications with MCMC and we hope, we can positively respond and implement the customized feature for the community.

1. There are some obvious features from other software like Tracer that could be duplicated in VMCMC to make it most useful. In particular, the ability to plot marginal distributions and correlations among different parameters probably could be important and interesting additions to VMCMC for accurate visual assessment of convergence and exploration of posterior distributions.

2. Displaying the ESS values for all ESS_{Max} estimates in a separate column in "Overview tab" could be helpful in analysing the convergence of each parameter.

3. An interesting feature could be having the tree marking functionality available on extracted portions of the trace plots. At this moment, only plotting tree markings on the full trace are supported.

4. It would also be outstanding if only those samples with particular topologies could be extracted directly.

5. If VMCMC could display plots of split frequencies in the way that AWTY does (and the way that the forthcoming RevBayes software will), that feature could really make the package more inclusive. The code is there and is being used for parallel chain analysis in calculating splits but it is not available for singular chains at the moment and will also require a new panel or pane to show it.

6. Select burnin from any of $\text{Gelman-Rubin}_{\text{Last Burnin}}$ or $\text{Geweke}_{\text{Last Burnin}}$ or $\text{ESS}_{\text{Last Burnin}}$ or ESS Normalized or ESS Standardized for the complete chain from command line. Currently the default burnin is calculated from $\text{ESS}_{\text{Last Burnin}}$ method and the only alternative is to supply a numerical value for burnin. There is no option to select other global convergence diagnostics.

7. Burnin estimators do not take advantage of comparisons between parallel runs to look for convergence on similar regions of parameter space, which seems like a lost opportunity for comparisons between parallel chains.

8. Make the tree analysis tab more interactive for example the selected topology should be highlighted in the 2D figure or becomes larger. At the moment it is hard to separate a specific topology from the other points around it and sometimes the header of each point is illegible.

14 FAQ

Q1) What software does VMCMC support?

A1) VMCMC supports MrBayes, PrIMe, JPrIMe and BEAST. For JPrIMe, VMCMC only supports DLRS and DLTRS at the moment. However, if the MCMC can be converted into tab delimited columns with the last column for newick formatted trees, then such a file can be analysed by VMCMC.

Q2) When I run VMCMC using other programs e.g. pDLRS, it runs fine but the convergence tests are always giving not converged. Why?

A2) VMCMC assumes that the first column of MCMC input to VMCMC is the iteration number, which is neglected and not not used in MCMC analysis. pDLRS has a repetitive column of iteration number, which can never converge and as ESSMax is VMCMC's current solution to estimating convergence, therefore the output from pDLRS can never converge. We are planning to add an option to the user, where he can specify to neglect particular column(s) for MCMC analysis and so presence of such a column can be handled by the program.

Q3) I get error message that seem to be related to the input format and which states that VMCMC input does not belong to the specified list of software (MrBayes, CODA, JPrIMe and PrIME). What could be wrong?

A3) This happens, when the parser is unable to parse MCMC input. Possible reason could be tinkering with headers in the files. Make sure that the original file output from software is provided to VMCMC. removing of samples is allowed but whole lines must be removed and in case of MrBayes, the tree and the numeric parameter files must correspond to each other. Also the Newick tree structure and Nexus tree structure must be maintained.

Q4) I get a warning that large number of trees are present in the posterior and then the Tree Analysis tab seems to be struck and not working sometimes. Why is that?

A4) You must wait for some time and the results will be displayed on the Tree Analysis tab. Such a thing occurs whenever the number of trees in tree posterior with greater than 0.2% frequency is more than 45. A warning is generated to the user since the 2D matrix computation for Robinson's Fould distance is very time consuming.

Q5) I am getting error message on screen related to parsing of numeric columns, when I give a single or parallel chain to VMCMC. Why is this error message generated?

A5) One reason for such a behavior is when values like INFINITY appear in numeric columns. The numeric data can not be parsed and an appropriate exception is raised for such a case. Make sure that the data is correct and numeric columns contain strictly numeric values.

Q6) I notice that the Graph panel takes a lot of time to load and almost all interactive functionalities (slider, burnin specification and parameter selection) are very slow on this tab. Is that a bug or natural behaviour of this tab?

A6) We did not notice this behaviour in Java SE 6. However, when VMCMC is run on Java SE 7, such a behaviour is observed. On a little investigation, Oracle mentions that the Java SE 7 has some significant delays in drawing 2D plots as compared to Java SE 6. Therefore, it is plausible that the reason for these delays are usage of Java SE 7 and not Java SE 6. That is why we recommend to run VMCMC on Java SE 6.

Q7) Does VMCMC support MrBayes with stationary trees (e.g. with just .p file)?

A7) VMCMC supports MrBayes files (.p with .t as well as just .p) files so MRBayes runs with stationary/fixed tree topology is supported. Note that we can even open .mcmc file of MrBayes, which is a tab separated file but which do not correspond to parameter posterior samples.

Q8) Whereas log files can become large, that's generally not a real problem in terms of memory management. The same can not be said about output files containing tree samples. Should some advice be given concerning the magnitude of .trees files (in terms of the number of sampled trees)?

A8) If the output file contains very large number of samples (e.g., more than tens of thousands of samples) or if not enough memory is available for VMCMC, the processing speed of VMCMC becomes very slow. However, the progress bar has been implemented exactly to show that VMCMC is working and has not crashed or gotten stuck. However we have tried with MCMC trace file generated from JPrIMe with one hundred thousand very large trees (with more than 200 leaves) and the response time is about 20 seconds to load the complete file. So we would not like to limit the number of sampled iterations but can say that waiting time increases exponentially with the number of sampled iterations and one should thereby expect longer waiting times.

Q9) Why are "ESS Normalized" and "ESS Standardized" not displayed when the GUI is loaded for an MCMC file? Is it a bug?

A9) "ESS Normalized" and "ESS Standardized" are joint burnin estimators and convergence diagnostics and take a long time to compute. This computation is being done using a threaded architecture and we wanted to show the other results right away without waiting for these computations. Therefore, we load the GUI as soon as the file is read and other computations are done but leave the two joint ESS estimators blank until the values have been computed. This is particularly noticeable if a MCMC file with large number of samples is provided. So it is not a bug but an intended feature.

Q10) For MrBayes output, it is very confusing that the default files that can be opened are *.mcmc files. This mislead me to open the *.mcmc output from a MrBayes run which gave completely nonsense results

A10) MCMC tools like PrIMe and JPrIMe have used the .mcmc suffix for their output and VMCMC was specifically designed for most popular MCMC tools. Therefore, one should open .p file for MrBayes and avoid opening .mcmc file.

Any further questions, please send to the corresponding authors.

15 Team

As one of the greatest writers of 20th century Helen Keller says "Alone we can do so little; together we can do so much", we would like to introduce the team behind VMCMC in the following image.

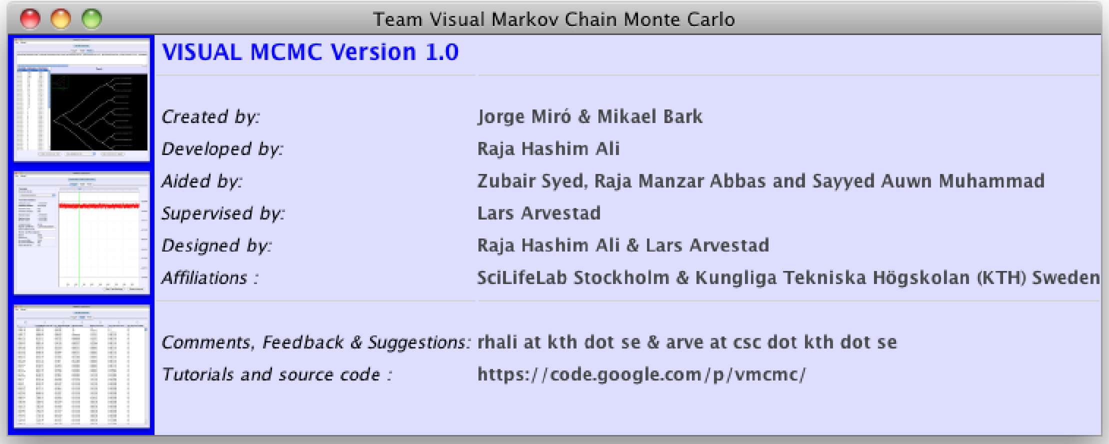

Supplement: Additional file 4 — Tutorial file containing all information about input, output, commands to run VMCMC from command line, GUI options, FAQ and even a list of features we have identified as interesting and which we will target for implementation in future. It is in pdf format and can be open using Adobe Acrobat Reader. (PDF 1710.08 KB) [file 12859_2017_1505_MOESM4_ESM.pdf]
